# Exploring Drivers of Binge Eating in Individuals With Food Insecurity and Recurrent Binge Eating: A Qualitative Analysis

**DOI:** 10.1002/eat.24434

**Published:** 2025-04-02

**Authors:** Emilie A. Green, Kristin L. Schneider, Angela Chang, Brian A. Feinstein, Isabel R. Rooper, Jennifer E. Wildes, Andrea K. Graham

**Affiliations:** ^1^ College of Health Professions Rosalind Franklin University of Medicine and Science North Chicago Illinois USA; ^2^ College of Arts and Sciences Suffolk University Boston Massachusetts USA; ^3^ Center for Behavioral Intervention Technologies Northwestern University Feinberg School of Medicine Chicago Illinois USA; ^4^ Department of Medical Social Sciences Northwestern University Feinberg School of Medicine Chicago Illinois USA; ^5^ Department of Psychiatry & Behavioral Neuroscience The University of Chicago Chicago Illinois USA

**Keywords:** binge eating, feast‐or‐famine, fluctuating food access, food insecurity, obesity, stress

## Abstract

**Objective:**

This study explored the drivers of binge eating in people with food insecurity and recurrent binge eating.

**Method:**

Participants were 28 adults, ages 26–69 (*M* = 46.22, SD = 11.94; 64.3% female), who endorsed current food insecurity and recurrent binge eating (≥ 12 episodes in the past 3 months). Individual semi‐structured interviews were conducted to assess the relationship between food access and binge eating. Data were analyzed using thematic analysis.

**Results:**

Participants reported binge‐eating drivers unique to individuals with food insecurity (fluctuating food access) and drivers commonly observed in those who binge eat, such as binge‐promoting narratives and drivers related to mental health (e.g., stress) and physical health (e.g., sleep deprivation). The themes “fluctuating food access” and “negative impacts of mental and physical health” also interacted to promote binge eating, such that financial uncertainty promoted stress, which led to binge eating.

**Discussion:**

This qualitative assessment of individuals' lived experience with food insecurity and binge eating suggests the relevance of considering food insecurity‐specific factors, mental and physical health, and binge‐promoting narratives when addressing binge eating in this subpopulation. Future research should explore whether improving access to nutritious foods and enhancing coping strategies address binge eating in individuals with food insecurity.

**Summary**

- Although food insecurity is associated with binge eating, the mechanisms of this relationship are unclear.
- We interviewed adults with food insecurity and binge eating to learn what drove their binge eating.
- Drivers included fluctuating food access, mental and physical health, and binge‐promoting narratives.
- Results suggest that assessing food insecurity in eating disorder treatment, targeting financial stress in interventions, and improving nutritious food availability may reduce binge eating in this subpopulation.

## 1 Introduction

Food insecurity is characterized by an inability to make available food last or to afford balanced meals (United States Department of Agriculture, Economic Research Service 2012). Affecting both physical (e.g., hypertension, type 2 diabetes; Seligman et al. 2007) and mental health (e.g., depression, anxiety; Pourmotabbed et al. 2020; Reeder et al. 2022; Wolfson et al. 2021), food insecurity has been associated with binge eating, binge‐eating disorder, and eating disorder pathology (Rasmusson et al. 2019; Becker et al. 2019; Hazzard et al. 2020; Stinson et al. 2018). Even at sub‐clinical levels, binge eating has adverse health consequences, including metabolic syndrome and weight gain, as well as depression and other mental health problems (Roehrig et al. 2009). Given the adverse health consequences associated with food insecurity and binge eating independently, their co‐occurrence is a significant public health issue. However, the mechanisms that explain their relationship are relatively unknown.

Models have been proposed to explain the association between binge eating and food insecurity. For example, the feast‐or‐famine model theorizes that monthly food subsidies and other financial resources (e.g., monthly paychecks) contribute to a cycle in which individuals alternate between periods of food abundance and restriction (Dinour et al. 2007). In this model, binge eating increases when food access is abundant. Then, as finances become more limited over the course of the month, individuals restrict their intake to make food last longer, thereby increasing their risk of binge eating when money for food becomes available again (Dinour et al. 2007; Hamrick and Andrews 2016; Hazzard et al. 2023). Alternatively, the affect regulation model posits that individuals binge eat to alleviate negative emotions (Haedt‐Matt and Keel 2011). Since food insecurity is associated with heightened stress (Kosmas et al. 2023), individuals with food insecurity may be more vulnerable to negative emotions and subsequent binge eating (Walker et al. 2019). A third model indicates that the relative affordability of energy‐dense foods could contribute to binge eating. Binge episodes typically comprise energy‐dense, palatable foods (Rosen et al. 1986), which can disrupt appetite control by promoting the desire to eat and overriding the body's satiety cues (Appelhans et al. 2011). By comparison, lower‐density foods (e.g., fruits and vegetables) tend to cost more, making them harder to access.

Research supporting these models in individuals with food insecurity is limited. Only one study has prospectively assessed the relationship between food access and binge eating and found support for the feast‐or‐famine cycle (Hazzard et al. 2023). Additionally, all but one of the prior studies on food insecurity and binge eating are limited by their use of self‐report questionnaires, which rely on pre‐set response options. Self‐report questionnaires preclude deeply understanding individuals' lived experiences, which could help inform and validate the theoretical models that explain individuals' experiences. Further, overreliance on self‐report questionnaires is problematic because they were originally created and validated in predominantly affluent samples (Hazzard et al. 2020) and thus may not accurately assess binge eating among people with food insecurity.

By contrast, qualitative assessment offers an ideal methodology to more comprehensively understand individuals' lived experiences. To our knowledge, only one study has used using qualitative methods to investigate the relationship between food insecurity and binge eating in adults. Frayn et al. (2022) interviewed 14 adults with past or present food insecurity (93% female; 50% with current food insecurity) who had completed individual outpatient treatment for binge eating. Findings suggested that variable food access increased stress and exacerbated the binge‐restrict cycle in those with food insecurity, which promoted and maintained binge eating, and that having current food insecurity interfered with treatment. While notable, assessing this relationship following treatment may have impacted people's understanding of their binge eating and relationship with food. Assessing these constructs in a treatment‐seeking sample could augment our field's understanding of this relationship. Moreover, Frayn et al. (2022) focused on how participants perceived food insecurity impacted their binge eating, but did not examine the broader drivers of binge eating in those with food insecurity. Therefore, additional qualitative research is needed to explore the factors that drive binge eating among individuals with food insecurity, to inform theoretical models and support intervention development.

The present qualitative study broadly examined drivers of binge eating in adults with food insecurity and recurrent binge eating, toward comprehensively understanding the complex interplay between food insecurity and binge‐eating behaviors. Based on existing literature, we hypothesized that the drivers of binge eating would align with three proposed mechanisms: variable food access, alleviating negative emotions, and affordability of energy‐dense foods.

## 2 Methods

### 2.1 Participants

Participants were 28 non‐pregnant, English‐speaking adults (≥ 18 years), with obesity (BMI ≥ 30 kg/m^2^ based on self‐reported height and weight), recurrent binge eating (≥ 12 episodes in the past 3 months, plus distress), and food insecurity (≥ 2 on the 30‐day six‐item short form food security survey; United States Department of Agriculture, Economic Research Service 2012). Binge eating was assessed via questions from the Eating Disorder Diagnostic Scale (EDDS; Stice et al. 2000). Individuals were not required to meet criteria for binge‐eating disorder or bulimia nervosa, as this study explored how binge eating behaviors relate to food insecurity. (Compensatory behaviors were not an exclusion criterion, and were assessed with the EDDS following the interview.) Because we were interested in designing a digital intervention to address binge eating in this population, eligible individuals also had to be interested in losing weight and reducing their binge eating, and have a smartphone and e‐mail address to reflect a target user of the digital intervention.

Sample characteristics are in Table 1. Participants had a mean age of 46.22 years (SD = 11.94, range = 26–69). Sixty‐four percent identified as female, 25% male, 7% a different gender, and 4% (*n* = 1) not reported. Regarding race, participants identified as White (53%), Black (39%), 4% other (*n* = 1), and 4% (*n* = 1) preferred not to answer. Regarding ethnicity, participants identified as non‐Hispanic (79%), Hispanic/Latino (18%), and 3% preferred not to answer (*n* = 1). All participants had food insecurity, with 60.7% having low food security and 39.3% very low food insecurity. At screening, participants' mean BMI was 39.8 (SD = 6.02; range = 30.5–52.8) and the mean number of monthly binge episodes was 9.17 (SD = 4.20; range = 4–16). Just over half (55.6%) endorsed engaging in ≥ 12 episodes of a compensatory behavior in the past 3 months, the most common of which was fasting. The median annual household income was $30,000. The sample had a mean (SD) income of $31,195 ($18,098). Thirty‐five percent of participants reported living alone. Household size ranged from one to six people (including self), with individuals living with an average of 1.48 additional household members.

**TABLE 1:** Participant demographics.

ID	Sex	Age	Race and ethnicity	Food security level
1	Male	33	White and Hispanic/Latino	Very low
2	Female	41	White	Low
3	Female	56	Black or African American	Low
4	Female	44	Black or African American	Low
5	Female	58	White	Very low
6	Male	42	White	Very low
7	Female	50	White	Low
8	PNTR	PNTR	PNTR	Very low
9	Male	45	Black or African American and Hispanic/Latino	Very low
10	Female	63	White	Very low
11	Female	24	Black or African American	Very low
12	Male	52	Black or African American	Low
13	Female	60	White	Low
14	Female	29	Black or African American	Low
15	Female	41	Black or African American	Very low
16	Male	58	Black or African American	Very low
17	Female	37	White	Very low
18	Female	50	Black or African American	Low
19	Other	69	White	Very low
20	Non‐Binary	26	White	Very low
21	Female	34	White	Very low
22	Male	37	White	Low
23	Female	51	White	Low
24	Female	51	Black or African American and Hispanic/Latino	Low
25	Female	40	Black or African American	Very low
26	Female	40	Other Race and Hispanic/Latino	Very low
27	Male	52	White and Hispanic/Latino	Very low
28	Female	65	White	Very low

*Note*: Food security level: low food security = score of 2–4; very low food security = score of 5–6.

Abbreviation: PNTR, prefer not to respond.

### 2.2 Procedure

Data were collected between September and December 2020. Participants were recruited for a study on “overeating and difficulty accessing food.” Recruitment occurred online (e.g., social media) and via flyers in Chicago, Illinois. Interested individuals submitted an online screener. Eligible participants were invited to complete a 60‐min session, during which they verbally granted informed consent and completed a semi‐structured interview (~45 min), followed by questionnaires administered via REDCap (Harris et al. 2009, 2019). Each session was conducted via video call and audio recorded. Interviews were transcribed by Zoom and edited for accuracy by an approved research team member.

Participants received $20 for completing the assessment. Study procedures were approved by Northwestern University's Institutional Review Board.

#### 2.2.1 Interview Guide

The interview guide was developed by the principal investigator (AKG) and a medical student. Individuals were assessed on eating behaviors and experiences with limited food access over the past 28 days. Participants indicated notable events on a calendar, displayed via share‐screen for reference throughout the interview, consistent with the Eating Disorder Examination (Fairburn and Beglin 1994). Then participants were asked open‐ended questions about binge eating/overeating, undereating, and food access (e.g., how finances and affordability of food impact binge eating, similarities between the past month and months before the COVID‐19 pandemic). Appendix 1 includes the interview guide.

### 2.3 Analyses

Interview transcripts were analyzed using methods from thematic analysis (Braun and Clarke 2006). The research team comprised four raters: Two undergraduate students, a bachelor's‐level research assistant, and a clinical psychology doctoral student (EG). The principal investigator resolved discrepancies as needed. A detailed description of the analyses and steps to minimize bias is presented in Appendix 2.

Consistent with the philosophy of self‐reflexivity, the lead author acknowledges her position as an educated White woman whose perspective is limited by the absence of current food insecurity, obesity, or binge eating. Other coders encompassed a range of backgrounds, which remain undisclosed to respect their privacy.

## 3 Results

Fifty‐eight codes were included in the thematic analysis. The codes corresponding to each theme and subtheme are in Table 2.

**TABLE 2:** Reported drivers of binge eating in individuals with food insecurity.

Theme	Subtheme	Code	Example quote
Fluctuating food access *n* = 28 (100%)	Finances *n* = 28 (100%)	Decreased access to food (overall)	“When I have a lot of money, I tend to binge eat because I can get whatever I΄m craving at the time.” (P14)
		Healthy food unavailable	
		Coupons/sales, cheaper food options	
		Borrowing money for food purchases^a^	
		Food purchase when not enough money or in debt	“It΄s a pattern throughout the month. I find myself low on food at the end of the month. I don΄t binge when the food is scarce.” (P12)
		Increased access to food (overall) After paycheck^a^ Unhealthy food available (e.g., affordable)	“When I get money, I΄ll go buy a bunch of food and go straight into a binge…” (P22)
		Binge foods	“Even if I have a little extra money, I can justify [bingeing] to myself… I'm going to binge eat.” (P20)
		Prioritize food for self	
		Eating out	
		Lifestyle change impacting finances/food access	
		Unnecessary food purchases when money available^a^	
	Busyness *n* = 21 (75%)	Forgetting, busyness	“We're extremely busy all day long. It's nonstop from the moment I get up until midnight. Yesterday, I think what might have caused the binge was just finally getting a break to relax.” (P26)
		Poor meal planning	
		Night eating	
		Eating after work, evenings	
	Exposure to food cues *n* = 21 (75%)	Food purchase on impulse	“Seeing food commercials on TV, going to thegrocery store and seeing certain items in the aisle, like in their frozen food section – these are cues that can trigger a binge.” (P10)
		After grocery shopping/delivery	
		Lack of motivation/“willpower”	
		Availability of quick meals, convenient food	
	Misc. *n* = 23 (82%)	Access	“The week before I get food stamps, I get binge foods. When I get food stamps, I have money, and I buy healthier, non‐binge foods.” (P10)
		Uncertainty about food access	
		Undereating	
Negative impacts of mental and physical health *n* = 28 (100%)	Stress and negative emotions *n* = 28 (100%)	Stressed, overwhelmed, anxious Stressed about familial responsibilities Stressed about finances No appetite^a^	“I was stressed and the only thing that would make me feel better was bingeing” (P11)
		Depressed, sad, grief Worthless, feel like failure, hopeless Panic, scared	“I eat my feelings. So if I΄m feeling stressed or anxious or anything, then I eat.” (P5)


		Shame, guilt	“When I binge, I hide away. I don't answer my phone. I don't talk to anybody. I isolate alone in my room because I don't want my roommates to see how much I'm eating or because I feel embarrassed. I shut everybody out even though I already feel isolated. I'm just contributing to the problem, which I know is not conducive for recovery.” (P20)
		Bored (e.g., free time)	
		COVID	
		Mood shifts	
		Interpersonal problems^a^	
		Feeling isolated	
		Judgments/pressure from others^a^	“My anxiety makes me not want to eat but then when I'm feeling better, I eat more.” (P1)
		Judgments from self, poor body image	
		Comparisons (to past self, to others)^a^	“When I'm stressed, eat snacks: cheese and crackers, chips, cheese dip. I eat until they're gone.” (P5)
Eating in secret^a^			
Eating alone^a^			


		Temporary gratification, relief, relaxation, distraction	
Snacking/nibbling/grazing when stressed		
Celebration, excitement	

	Urges *n* = 17 (60.7%)	Feeling compelled to eat Craving Addiction	“It doesn΄t matter how full you get, you may not be hungry, but it΄s like you have to have a sweet.” (P17)
	Physical health *n* = 15 (53.6%)	Lack of sleep^a^	“I sleep so little, maybe 2–4 h when working. I'm so pooped, so I'm always stressed. If I do eat it's something sweet.” (P7)
		Low energy, tired	
Physical health			
Binge‐promoting narratives *n* = 12 (42.9%)	—	Rationalizing the binge	“At 278 lbs., how am I going to lose 150 lbs.? I'm never going to be able to, so why not just eat? What does another three to five pounds matter? What's the difference between 275 and 280?” (P13)
		Compensating for the binge	
		After diet	
		Before diet	
		Childhood perceptions, fear of being hungry	

*Note*: Percentage values represent the percentage of participants endorsing this theme/subtheme.

^a^ Consistent with standards for reporting inconsistent responses in qualitative research, we report codes within themes that were infrequently applied, which we defined as endorsed by three or fewer participants.

### 3.1 Binge Eating

During the interview, binge eating was defined as “a time when you ate a large amount of food and felt like you could not control what or how much you were eating.” If during the interview participants used different language to describe their binge eating, interviewers aligned with their language to maintain rapport. After defining binge eating for participants, they were asked to describe their binge episodes. One participant shared, “I can eat what you might think was three meals. And then, as soon as the discomfort goes away from my being so, so full, I'll start eating again” (P13). Another participant described, “At first, it doesn't really start out with the intention to binge. It's more like searching for comfort except, then you can't stop eating” (P8).

### 3.2 Undereating

During the interview, undereating was defined as “when you eat less food than usual or eat less food than you would like to eat.” Participants described how undereating episodes contribute to binge eating, stating, “On days where I eat less overall during the day, this usually looks like very little or no breakfast or lunch, I eat dinner but I'm still pretty hungry. Then I go back for more and the rest of the night respond to the food cravings” (P1). Another said, “Binges usually happen on days that I don't get a chance to eat throughout the day. I just get these sudden urges of wanting to eat large amounts late at night” (P26).

While most participants described undereating due to food access issues, in one case (3.57%), a participant reported undereating due to stress: “Sometimes my anxiety causes me not to eat. I don't feel hungry. But when I'm feeling better, I eat more” (P1). Because of the role of stress here, this response was categorized under the *stress and negative emotions* subtheme.

### 3.3 Fluctuating Food Access

All participants (100%) described *fluctuating food access* as a driver of binge eating. In general, participants described binge eating during periods of increased food access, followed by undereating as food became scarcer. To illustrate the binge‐restrict cycle, one participant described, “Being without food makes the bingeing worse because once I get a hold of some food—I'm going to overeat. I have to go into a binge” (P18). Participants cited multiple reasons for their *fluctuating food access*, including structural barriers (i.e., policies/systems reducing food access) and external (immediate, tangible) barriers that limited consistent access to food.

While our initial questions focused on the economic aspects of food access (i.e., insufficient money to purchase food), participants' responses revealed a more complex picture. Three subthemes appeared to influence binge eating resulting from *fluctuating food access*: *finances*, non‐economic factors such as time constraints (*busyness*), and *exposure to food cues* (when food is accessible).

All participants discussed how *finances* led to *fluctuating food access* and promoted binge eating. Generally, participants reported binge eating during times of greater financial abundance due to their ability to purchase food. One participant stated, “When I have a lot of money, I tend to eat a lot because I can get anything I'm craving at that time” (P14). Some participants described binge eating during periods of relative financial constraint, due to factors like stress. Participants also described consuming less food to prioritize essentials (e.g., rent), which led to binge eating when food was available later. Participants described how having money available led them to make food purchases they deemed unnecessary (e.g., buying less nutritious foods that they perceived they “didn't need”), which unintentionally resulted in binge eating by making binge‐promoting food available. Participants also reported purchasing energy‐dense foods due to their relative affordability, which participants prioritized over food quality. This promoted binge eating. For example, one participant commented, “Healthy eating isn't cheap and it's easier to get a bag of chips than vegetables, and that's what you're going to be binge eating later on. When you're buying groceries and you have a very small budget, you have to buy what can feed you for longer, not healthier” (P8).

Most participants (75%) described how the subtheme of *busyness* led to *fluctuating food access*. Some were unable to access food for extended periods due to work or other responsibilities. This drove binge eating at night and after work. Relatedly, *busyness* during the day led to poor meal planning (which made food access inconsistent) and forgetting to eat. One participant explained how *busyness* prompted binge eating, stating, “Working a 12‐hour shift, I don't get time to eat […] so after work I'm very hungry” (P6). Finally, most participants (75%) described how the subtheme, *exposure to food cues*, at home or in their daily lives triggered binge episodes. One participant said, “I try not to have binge foods on hand. If I've got it on hand, I have very little willpower” (P19). Individuals also reported purchasing binge foods on impulse when walking by them in the grocery store or binge eating when food was available following grocery shopping or food delivery.

### 3.4 Negative Impacts of Mental and Physical Health

The second theme endorsed by all participants was using food to provide relief from the *negative impacts of mental and physical health*. All participants (100%) described how binge eating was prompted *by stress and negative emotions* such as depression, boredom, feeling hopeless or worthless, anxiety, panic, and mood shifts generally. *Stress*, the principal subtheme, was the most prevalent negative experience reported. One specific type of stressor was social stress (e.g., familial responsibilities, interpersonal problems, isolation). For example, related to isolation, a participant said, “When I binge, I hide away. […] I already feel isolated, so hiding is just contributing to the problem” (P20). Another stressor that led to binge eating was the COVID‐19 pandemic, for example, due to taking on additional responsibilities at home or feeling anxiety about contracting the disease. One participant described, “I have a teenage grandson. He stayed with me for two weeks; it's his senior year. They're doing the online thing. He's behind on everything, and I was made to feel like it was my fault […] so I was constantly eating” (P28). Indeed, many participants described how the pandemic exacerbated their day‐to‐day stressors.

About half of participants (54%) described *physical health*‐related factors that led to binge eating. *Physical health* factors included sleep disturbances, hormonal fluctuations, diseases, and physical discomfort or injury. For example, one participant explained how their health problems made them feel stressed and then binge: “I had health stressors like rheumatoid arthritis and sciatica. I'm constantly going to the doctor and the ER [emergency room]. Also, I just learned two weeks ago that I'm diabetic, type two. So that's another stressor from all the complications that came after getting COVID” (P24). Finally, over half of participants (61%) said that their binge eating was driven by the negative experience of *urges*, which they characterized as feeling compelled to eat. In fact, some participants characterized themselves as addicted to food: “I binge eat because I'm a food addict; I have an eating disorder, and these foods give me a great deal of pleasure” (P10). Another participant said binge eating replaced their alcohol addiction; “It used to be that I'd drink, but now I eat” (P16). Other participants emphasized the intensity of the *urges* and described the attempts to curb them as driving their binge eating. As one participant explained, “You'll tend to eat something sweet, but then you crave something else and then you eat that, and then that leads to craving something else” (P28).

### 3.5 Binge‐Promoting Narratives

The third theme comprised *binge‐promoting narratives*, which refer to beliefs that participants use to explain their binge eating. Nearly half of participants (43%) reported *binge‐promoting narratives*. For example, participants said binge eating occurs because of lifelong patterns involving food that make binge eating feel inevitable, with one person noting, “I've always been that way through my whole life, it's an eating pattern I've always had. It's tough to break away from” (P27). Participants also reported rationalizing binge eating through the promise of future dieting. For example, a participant said they rationalized binges by explaining that “I've already blown it; why not just go ahead and have everything I want? And then I'll start fresh again tomorrow” (P13). Finally, some participants rationalized binge eating in response to failing to adhere to a diet. One participant described, “When I messed up on the keto diet, I decided that I was going to eat everything in sight” (P4).

### 3.6 Connections Between Themes

Connections were observed between the themes of *fluctuating food access* and *negative impacts of mental and physical health*, with 103 instances of overlap. The total occurrences of codes within the *fluctuating food access* and *negative impacts of mental and physical health* themes were 280 and 249, respectively. In 36.8% of instances where *fluctuating food access* was mentioned as a driver (280 total instances), *negative impacts of mental and physical health* were also coded. Similarly, in 41.4% of instances where *negative impacts of mental and physical health* were mentioned as a driver (249 total instances), *fluctuating food access* was also coded.

The *stress and negative emotions* subtheme (227 total excerpts) overlapped with each of the subthemes of *fluctuating food access*: most frequently with *finances* (30%; 68 excerpts), followed by *exposure to food cues* (8%; 19 excerpts), and *busyness* (8%; 18 excerpts). Participants described how binge eating episodes that occurred during periods of decreased food access often resulted from stress related to uncertainty about accessing food when money was tight. One participant said, “Because we didn't know if we would have enough money to get Thanksgiving food, I binged a lot” (P24). Participants reported similar experiences when paying for essentials: “If there's going to be any time that I would eat more, it would be around when it was getting near when I had to come up with a power bill, which I skid through every month” (P7). Participants also described how binge eating negatively affected their *finances* and *stress*. One participant said, “I ordered so much takeout and really screwed myself over financially for the rest of the week” (P20).

Regarding the interplay between *busyness* and *stress and negative emotions*, participants explained that their work schedules promoted undereating during the day, and this triggered binge episodes after work and in the evenings due to both access to food and a chance to alleviate stress. *Exposure to food cues* also drove binge eating in connection with *busyness* and *stress and negative emotions*. One participant explained, “I was really stressed and had two tests. I didn't have time to cook. I remember bingeing on the Halloween candy outside our dorm room because it was the only thing that made me feel less overwhelmed” (P11). Substantial patterns of overlap were not observed between the third theme, *binge‐promoting narratives*, and any other themes, as it only overlapped with 16 excerpts in *fluctuating food access* (5.7% of 280 total excerpts) and 17 excerpts in *negative impacts of mental and physical health* (6.8% of 249 total excerpts).

## 4 Discussion

This study explored perceived drivers of binge eating among individuals with food insecurity and recurrent binge eating. As hypothesized, themes and subthemes aligned with theoretical models explaining the link between binge eating and food insecurity. The fluctuating food access theme was consistent with the feast‐or‐famine model, as participants generally reported undereating when finances were tight, and binge eating when financial resources were distributed (e.g., paychecks, food assistance benefits). Our results also supported the hypothesis that individuals with food insecurity binge eat relatively affordable, highly palatable foods (e.g., candy, chips). The “binge foods” code often consisted of descriptions of energy‐dense foods, and codes of “unhealthy food available” and “coupons/sales, cheaper food options” indicated how the availability of less nutritious, more affordable, energy‐dense foods led to binges. Participants reported buying these foods because they were more palatable and affordable than nutritious options. Finally, the stress and negative emotions subtheme, supported the affect regulation model, as participants reported binge eating to cope with stress and negative emotions associated with food insecurity (e.g., uncertain food access, financial stressors). Additionally, we observed connections among heightened stress, cyclical restriction, and the relative affordability of energy‐dense foods that may exacerbate binge eating among individuals with food insecurity.

Our findings replicate and extend the qualitative findings by Frayn et al. (2022), which also identified stress and fluctuating food access as key drivers of binge eating in populations with food insecurity. However, our larger sample size (28 vs. 14 participants) of treatment‐seeking individuals (vs. those completing outpatient treatment) coupled with our broader focus on all drivers of binge eating enabled us to uncover a variety of contributing factors. For example, we identified influences beyond stress that contribute to binge eating, such as other mental‐ and physical health‐related factors and urges. We also identified factors that drive eating in the context of fluctuating food access that were financially related (e.g., binge eating during periods of financial abundance, undereating to prioritize essential expenses, and making unnecessary food purchases when resources allowed) as well as non‐financially related (e.g., busyness, exposure to food cues). Another contribution is our identification of the interplay between the impacts of mental and physical health and fluctuating access to food, which exacerbate binge‐eating behaviors. Additionally, we identified specific binge‐promoting narratives, such as viewing binge eating as a result of lifelong patterns or thoughts that serve to rationalize binge eating.

These findings underscore the utility of assessing food insecurity when treating individuals with binge eating so that these interrelated issues can be addressed in treatment, especially as food insecurity can interfere with eating disorder treatment and recovery (Frayn et al. 2022). Indeed, some of our findings align with existing treatment models for binge eating (e.g., stress and negative emotions as intervention targets in cognitive behavioral therapy (CBT) (Fairburn 2008) and interpersonal psychotherapy (IPT; Wilfley et al. 1993)). However, other drivers found here indicate that intervention tailoring may be required to improve treatment efficacy in individuals with comorbid food insecurity. For example, neither CBT nor IPT address financial barriers to stable food access that can lead to binge eating. Treatment adaptations, such as providing financial support, may therefore be beneficial. Interventions to improve coping in response to the negative impacts of mental and physical health, especially financial stress, in this population may be beneficial as well. Our findings also present clinical and policy implications and suggest that providing financial benefits, such as via the Supplemental Nutrition Assistance Program, on a more consistent schedule (e.g., weekly instead of monthly) could address some drivers of binge eating among individuals with food insecurity by stabilizing their access to food.

### 4.1 Limitations

Our study adds to the growing literature on the lived experience of binge eating among people with food insecurity; however, limitations should be noted. Our study is subject to the limits of self‐report and relies on retrospective accounts of binge‐eating drivers, which may be subject to recall bias (Schwarz and Sudman 1994). Data collection occurred during the COVID‐19 pandemic, which may have impacted our results, given known impacts of the pandemic on food access and eating disorder psychopathology and treatment (Devoe et al. 2023). Indeed, some participants' responses aligned with some of the known impacts of COVID‐19 (i.e., stress related to mandates, uncertainty about potential long‐term health effects of COVID, lifestyle disruptions); however, many (though not all) participants indicated that the impacts of COVID‐19 had not substantially changed their binge eating. These findings remain relevant post‐pandemic because the negative impacts on mental and physical health, fluctuating food access, and binge‐promoting cognitions remain relevant to binge eating regardless of the source. To that effect, the opportunity to examine coping and eating behaviors ecologically during a period of heightened, widespread stress is one strength of this study. Another limitation is that the validity of these results was not checked with participants following completion of the analysis. Generalizability of these results may be limited due to our predominantly female sample. Study eligibility criteria also included having obesity and a desire to lose weight and reduce binge eating; therefore, findings are specifically applicable to those with recurrent binge eating who are in larger bodies and are also interested in losing weight. While it is common for individuals with binge eating to present to psychological treatment with a goal of losing weight (Brody et al. 2005; Voss et al. 2023), these research questions should be explored among people with binge eating and food insecurity of varying body sizes and preferences for weight change, as well as with an expanded sample that includes broader racial, ethnic, and gender diversity. A related limitation is that all participants were interested in losing weight, but their motivations for weight loss were not assessed, such that it is not possible to determine the number of individuals who restricted their food intake due to shape/weight concerns, food insecurity, or both. Additionally, data on the history of food insecurity or binge eating were not collected, which limits our understanding of how these factors impact current binge eating. Future research might explore the influences of past food insecurity on binge eating.

### 4.2 Conclusions

This qualitative study showed that binge eating in individuals with food insecurity is driven by fluctuating access to food (i.e., the feast‐or‐famine cycle and the availability of relatively affordable energy‐dense foods), negative impacts of mental and physical health (e.g., stress, negative emotions, urges, physical health), and binge‐promoting narratives (e.g., perceptions from childhood and rationalizations for binge eating). These drivers promote binge eating independently and synergistically. While some drivers were not unique to those with food insecurity, such as mental and physical health factors and binge‐promoting narratives (Stein et al. 2007; Haedt‐Matt and Keel 2011; Legenbauer et al. 2018; Kenny et al. 2018), fluctuating access to food was identified as unique to those with food insecurity, indicating areas for intervention adaptation. Indeed, providing stable, increased access to food could help address all of these drivers, though future research must test if this improves binge eating. Given how stress and negative emotions impact binge eating, interventions should also offer strategies to enhance coping in response to stress.

## Author Contributions

**Emilie A. Green:** formal analysis, methodology, validation, writing – original draft, writing – review and editing. **Kristin L. Schneider:** formal analysis, methodology, supervision, validation, writing – review and editing. **Angela Chang:** data curation, formal analysis, investigation. **Brian A. Feinstein:** methodology, validation, writing – review and editing. **Isabel R. Rooper:** validation, writing – review and editing. **Jennifer E. Wildes:** writing – review and editing. **Andrea K. Graham:** conceptualization, data curation, formal analysis, investigation, methodology, resources, supervision, validation, writing – review and editing.

## Conflicts of Interest

The authors declare no conflicts of interest.

## Data Availability

The data that support the findings of this study are available from the corresponding author upon reasonable request.

## Appendix 1 Interview Guide

The following guide reflects the types of questions that will likely be asked of participants during the interview. Some items may change due to the iterative nature of qualitative research. However, the overall topic and level of sensitivity will remain consistent.

### Introduction

The reason we are talking today is I want to learn about your eating habits and how they may relate to problems you have accessing food. I am interested in different types of eating behaviors you may have. I am also interested in how they may be affecting your eating. I will ask you different questions, and there are no right or wrong answers. My goal today is to learn about *your* experiences. I may write some things down while we talk so I can look at them later. Before we start, I am going to start recording this zoom call/turn on the audio recorder. Do you have any questions before we start?

To begin, I am going to show you a calendar of the past 28 days, which is about the past month. It ends on yesterday because today is not over yet. So it goes from yesterday (day and date) to (day and date).

1. What I would like you to do now is tell me about any events that have happened in the past 28 days since this will help us discuss these 4 weeks. Then we can note these down on the calendar. I know this will test your memory because the weeks tend to blend together. To help with this, I have already noted down the holidays. Have there been any events out of the ordinary, such as celebrations of any type, trips away, or days off work?
  - Are there times in the past month when you were more likely to binge or overeat? As a reminder, a binge is a time when you ate a large amount of food and felt like you could not control what or how much you were eating. So when were these times, and what kinds of things were happening?
    - Which days did you overeat or have a binge?
  - Now looking at the calendar, can you tell me which days money was tight?
    - Did you ever receive food stamps? If so, which days?
    - Do you ever receive coupons? If so, which days?
  - Which days did you have enough money to buy food?
    - (We are going to talk more about this later, but how do you think that money affects your overeating?)
  - Are there any times you under‐ate? Undereating is when you eat less food than usual or eat less food than you would like to eat.
    - Are there times in the month when you were more likely to undereat? When were these times, and what kinds of things were happening?
    - Which days did you undereat?
  - Do other months look a lot like this one, or was this one different?
    - Would you say that this month was similar to other months *before* COVID? (yes or no)

Thank you for filling out this calendar with so many details. I am going to keep it up on the screen for the rest of the interview to help you answer other questions.

2 I would like to talk more about times when you overate or had a binge.
  - Tell me about a time this recently happened. Describe what happened. What you were doing and feeling before it began? What were your feelings afterward?
  - What types of things cause you to overeat or binge (“triggers”)? For example:
    - Feeling stressed or upset?
    - Being around foods that taste good or that you really like?
    - Being very hungry?
    - Feeling unsure of when you will eat *these* foods again?
    - Feeling unsure of when you will eat again?
    - Wanting to eat these foods before someone else does?
    - Having increased access to food?
  - When do you tend to overeat or binge eat? (time of day)
    - Start of the month? End of the month?
    - Are these times related to any specific event or day?
  - What foods do you tend to eat? Why do you pick those foods?
  - How does money impact your binge eating? How does binge eating affect your finances?
    - When money is tight, how do you prioritize what to spend money on? How do you make decisions about food when money is tight, such as:
      1. Do you eat different foods when money is tight?
      2. Does your spending on foods change when money is tight?
      3. Do your eating patterns change when money is tight
3 Are you worried about your overeating or binge eating? Tell me about the types of worries you have. In what ways might your binges cause problems for you?
  - weight gain
  - physical and/or mental health?
  - ability to take care of things at home or work?
  - relationships with other people?
  - finances?
4 Have you tried to cut back on your binge eating? What did you try? How did you learn about what things to try? What worked? What got in the way of keeping that going?
  - What makes it hard to cut down on your binge eating?
  - Who or what made you want to cut down on your binge eating?
  - Has anyone helped you try to cut down on your binge eating?
5 If you could have access to an online program that could help you reduce your binge eating and lose weight:
  - What would make it likely for you to do this program?
  - What could get in the way of you doing this program?
    - Financial concerns (e.g., cost of program, limited WiFi or data plan)
    - Don't think my problems are severe enough
    - Providers will notunderstand
    - Don't feel ready
    - Don't think it will work for me
  - What types of things would be important to you in this program?
  - What do you think would be most helpful in your treatment?
    - Help with specific symptoms (e.g., binge eating)
    - Help with weight loss
    - Help with body image problems
    - Help with healthy eating and physical activity
    - Accountability from a professional or coach
6 Have you tried to lose weight recently? What have you tried doing? How did you learn about what things to try? What worked?
  - What makes it hard to lose weight?
  - What made you want to lose weight?
  - Has anyone helped you try to lose weight?

Thank you for answering these questions so thoughtfully; I really appreciate it. You are doing a great job thinking through these questions. Next, I'm going to switch gears to the next question.

7 Do you think others judge you because of your weight or your shape? Who makes these judgments? What judgments do people make about your weight/shape?
  - Do you make judgments about yourself because of your weight or your shape?
  - (If yes) Are these judgments about yourself based on things you see on the internet or on social media? (yes or no for first few interviews)
    - Inability to achieve a desired body shape as seen online
    - Inability to recreate healthy foods/recipes as seen online
8 (if participant has underate) Now I would like to talk about times you may undereat, which again means times when you eat less food than usual or eat less food than you would like to eat.
  - Tell me about a time recently when this occurred. What you were doing and feeling before and after?
  - What types of things cause you to eat less food (“triggers”)? For example:
    - Not having access to food?
    - Wanting to make sure others eat before you do (e.g., caretaking)?
    - Trying to control your weight or change your shape?
  - When do you tend to undereat?
  - What foods do you tend to eat during these times? Why do you pick these foods?
  - Some people describe having “stretch foods,” meaning foods that they buy to last longer when money is tight. Do you think of any foods as stretch foods? What foods are these? (ask this even if participant has not underate)
    - How long is the “stretch” period?
    - Tell me about a time when you experienced a “stretch” period?
9 Are you worried about your undereating? Tell me about the types of worries you have. In what ways might your undereating cause problems for you?
  - When did you experience undereating? How about periods of under‐eating?
  - Weight loss?
  - Physical and/or mental health?
  - Ability to take care of things at home or work?
  - Relationships with other people?
  - Finances?
10 Before we wrap up, what other things would you like to share that might be helpful for me to know about your eating?

Thank you so much for your time and thoughtful answers. As a reminder, the final activity is for you to complete a survey. (Research assistant) will send you a link to the survey in your email. Once you complete the survey, (research assistant) will email you the gift card. Do you have any other questions for me before we go? Thank you again for your participation and for completing the survey.

**Table:**

Calendar of the past 28 days						
(Week day)						
(Date)						
						
						
						(Yesterday)

## Appendix 2 Data Analyses

Interview transcripts were analyzed using methods from thematic analysis (Braun and Clarke 2006), which involved becoming familiarized with the data, generating initial codes, searching for, reviewing, and defining themes, and reporting results.

Following transcript review, the raters collaboratively generated a list of codes. First, all raters reviewed two transcripts together to align on the coding scheme. Then, raters independently coded the remaining transcripts and met in pairs to resolve any coding discrepancies. Unresolved issues were brought to the full team for consensus. The final codes were applied to the transcripts using the qualitative analysis software Dedoose (Sociocultural Research Consultants). Data were reviewed in full by the lead author to begin identifying themes. After initially constructing three themes, the lead author reviewed coded excerpts in Dedoose to ensure the themes adequately captured the source data. Subthemes were created by grouping codes within the overarching themes. Connections were noted between themes and subthemes based on excerpts that were double‐coded.

Several efforts were taken to reduce bias in question design and coding. To reduce bias in the interview guide, questions were grounded in existing literature. Additionally, the semi‐structured format was used to maximize the potential for participants to report on relevant topics while granting participants the flexibility to share their lived experiences. Participants were also asked to reflect on specific events in their lives and to describe both what happened and what they were feeling before, during, and after. Then, questions connected to these specific events were intended to minimize recall bias and encourage introspection and more detailed accounts.

During the analysis phase, we acknowledged the potential for confirmation bias in the lead author's (E.G.) and principal investigator's (A.K.G.) awareness of the study hypotheses. To mitigate this potential for confirmation bias, several strategies were implemented. All transcripts were anonymized prior to team review. The creators of the interview guide did not code the transcripts. The codebook was developed inductively from the data rather than from a pre‐existing coding frame or the researchers' analytic preconceptions, aligned with Braun and Clarke (2006). This allows themes to be identified organically from the transcripts. Though E.G. and A.K.G. were aware of study hypotheses, formulated based on their understanding of extant literature, codes were developed inductively and without a priori thresholds. This is consistent with Braun and Clarke's (2006) caution against imposing “rigid rules,” as they suggest that the number of instances or the proportion of representation within the data set does not determine the degree of importance. Before applying a code, coders closely examined the excerpt to determine whether the code represented participants' intended meaning in order to minimize the likelihood of adding their own interpretation to the data. Regular team meetings were conducted to discuss coding and discrepancies were brought to the full team for consensus. When grouping codes into themes, the lead author conducted the same authentication approach to ensure themes were anchored in participants'explicit statements. This approach, combined with double‐coding, helped maintain fidelity to participants' statements while minimizing interpretative bias.
